# Risk factors associated with urogenital schistosomiasis: a multilevel assessment approach using an Oversampling Schistosomiasis Survey (SOS) community-based, Plateaux region, Togo 2022

**DOI:** 10.1136/bmjph-2024-001304

**Published:** 2025-02-26

**Authors:** Smaila Alidou, Hélène E Kamassa, Fiali Lack, Essoham Ataba, Fiona M Fleming, Efoe Sossou, Manani Hemou, Kossi Yakpa, Mawèké Tchalim, Piham Gnossike, Penelope Vounatsou, Rachel Pullan, Katherine Gass, Ameyo M Dorkenoo

**Affiliations:** 1 Department de Médecine Sociale et Preventive, Université de Montréal, Montreal, Quebec, Canada; 2 Ministère de la Santé et de l’Hygiène Publique, Republique Togolaise, Lome, Togo; 3 Laboratoire de Microbiologie et de Contrôle de Qualité des Denrées Alimentaires, Unité de Recherche en Immunologie et Immunomodulation (UR2IM), Ecole Supérieure des Techniques Biologiques et Alimentaires (ESTBA), Université de Lomé, Lome, Togo; 4 West African Centre for Cell Biology of Infectious Pathogens (WACCBIP), University of Ghana, Accra, Ghana; 5 Department of Biochemistry, Cell and Molecular Biology, University of Ghana College of Basic and Applied Sciences, Accra, Ghana; 6 Laboratory Department of the Sylvanus Olympio Teaching Hospital, BP, Lomé, Togo; 7 National Malaria Control Program, Ministry of Health and Public Hygiene, Lomé, Togo; 8 Unlimit Health, London, UK; 9 Faculty of Health Sciences, Department of Biological and Basic Sciences, Université de Lomé, Lome, Togo; 10 Swiss Tropical and Public Health Institute, Allschwil, Switzerland; 11 Department of Diseases Control, London School of Hygiene & Tropical Medicine, London, UK; 12 The Task Force for Global Health, Decatur, Georgia, USA; 13 Ministère de la Santé de l’Hygiène Publique, Republique Togolaise, Lome, Togo

**Keywords:** Risk Assessment, Sociodemographic Factors, Prevalence

## Abstract

**Background:**

Urogenital schistosomiasis is endemic in Togo. Since 2010, Togo has used preventive chemotherapy to control the disease and periodically assess its impact. This study aimed to estimate the prevalence of urogenital schistosomiasis and identify associated risk factors among school-age children in three districts of the Plateaux Region of Togo.

**Methods:**

A cross-sectional study surveyed school-age children in three Togo districts, using an oversampling strategy of door-to-door visits to collect urine samples, metadata and lifestyle data. Statistical analyses, including descriptive and multilevel regression, were used to determine prevalence and investigate individual/community risk factors associated with urogenital schistosomiasis and infection intensity.

**Results:**

This study surveyed 6400 children, uncovering a 15.0% prevalence of urogenital schistosomiasis (95% CI: 14.1% to 15.8%). Notably, 48.3% (95% CI: 45.1% to 51.5%) showed heavy-intensity infections, averaging 38 eggs per 10 mL (range: 0–9688). Key risk factors included age (adjusted OR (aOR)=1.9), swimming in surface water (aOR=2.6) and residing in the Ogou district (aOR=11.2), while the Est-Mono district posed a lower risk (aOR=0.2). Factors such as gender, with boys at higher risk (aOR=1.7), age (aOR=2.9), school attendance (aOR=2.4) and swimming in surface water (aOR=4.7) were linked to infection intensity. Consumption of public tap water (aOR=2.4; 95% CI: 1.0 to 5.2) and residing in Ogou (aOR=28.6) increased intensity, whereas living in Est-Mono (aOR=0.0; 95% CI: 0.0 to 0.08) or using rainwater (aOR=0.0; 95% CI: 0.0 to 0.4) decreased it.

**Conclusions:**

The prevalence and intensity of urogenital schistosomiasis were found to be correlated with household and behavioural risk factors. Integrating these factors into national control programmes and improving access to safe water and sanitation facilities will be crucial in eliminating this disease as a public health concern in Togo.

**WHAT IS ALREADY KNOWN ON THIS TOPIC**

- Most previous studies on urogenital schistosomiasis in this context have relied on school-based sampling, excluding non-schooled children from the analysis. This approach limited the generalisability of findings, particularly concerning community-level dynamics.

**WHAT THIS STUDY ADDS**

- This study introduces a community-based sampling method that employs an oversampling approach, along with a multilevel analysis framework. This new methodology addresses the limitations of earlier studies by including both schooled and non-schooled children while considering contextual variables at the community level. Additionally, the methodology involves selecting 40% of villages in each district, ensuring they are nearly uniformly distributed across the geographical area of these districts to accurately reflect the real situation within each district.

**HOW THIS STUDY MIGHT AFFECT RESEARCH, PRACTICE OR POLICY**

- The findings suggest that community-based interventions, informed by multilevel risk assessments, are essential for mitigating the transmission of urogenital schistosomiasis. This evidence may encourage policy-makers to shift from solely individual-focused measures to more comprehensive strategies that address broader community factors.

## Introduction

 Schistosomiasis is a parasitic disease caused by several species of trematodes of the genus Schistosoma. It is prevalent in sub-Saharan Africa, South America, the Caribbean, the Middle East and Southeast Asia.^1 2^ In sub-Saharan Africa, which accounts for about 90% of global cases, prevalence among school-aged children was approximately 9.6% in 2019, with variation ranging from 0.5% in Rwanda to 32.6% in Guinea.^2^,^4^ The disease is also present, though less prevalent, in South America, the Caribbean, the Middle East and Southeast Asia. In these regions, prevalence rates vary but are generally lower than in sub-Saharan Africa, typically ranging from less than 1% to 25% in endemic areas.^5 6^ Infection primarily occurs when individuals come into contact with water contaminated with snails, which serve as intermediate hosts for these parasites.^2 4^ The predominant species in the African region are *Schistosoma haematobium* and *Schistosoma mansoni*, responsible for urogenital and intestinal schistosomiasis, respectively.^2^ These parasites rely on specific species of freshwater snails, such as *Bulinus* genus for *S. haematobium* and *Biomphalaria* genus for *S. mansoni.*^6^

Schistosomiasis is listed as one of the neglected tropical diseases by the WHO.^7^ Due to its morbidity and mortality, preventive chemotherapy (PC) through Mass Drug Administration (MDA) with praziquantel targeting the populations at-risk is the major strategy recommended by WHO to reduce the burden of the disease. Schistosomiasis ranks second among parasitic diseases, after malaria, in terms of socioeconomic importance and public health impact.^8^ It is estimated that over 200 million people worldwide are infected, and approximately 700 million people live in low-income and middle-income countries.^4 9^ More than 90% of infected individuals reside in sub-Saharan Africa, where it is estimated that this infection leads to approximately 70 million cases of haematuria, 32 million cases of dysuria, 18 million cases of bladder pathology and 10 million cases of major hydronephrosis.^4 10^ In addition to the high burden in Africa, countries in South America and the Eastern Mediterranean have also reported significant cases of infection, with approximately 2.25 and 17.27 million people affected in these regions, respectively. ^5 10^ This disease leads to nutritional deficiencies, stunted growth, decreased physical activity and poor academic performance, particularly affecting children.^9 11^

To reduce the adverse consequences associated with this parasitic disease, WHO urges all member states to regularly treat at least 75% of populations exposed to a significant risk of schistosomiasis-related morbidity.^12^ The recommended control strategy is currently being implemented in most African regions, including Togo since 2010.^13 14^ However, Togo faces significant challenges in access to clean water and adequate sanitation infrastructure. According to recent reports, only 63% of the population has access to improved drinking water sources, while 36.9% rely on unimproved sources.^15^ Open defecation remains a significant issue, with an estimated 43.32% of Togolese people defecating outside.^16^ These challenges increase the risk of waterborne diseases and negatively impact public health, particularly affecting vulnerable populations such as children.^17^ The predominant species in Togo are *S. haematobium* and *S. mansoni*,^18^ with *S. haematobium* more widespread in the country.^14 19^ In Togo, as in other African countries, Bulinus genus acts as the primary intermediate host *for S. haematobium,* while Biomphalaria genus serves as the main host for *S. mansoni*.^6^ In 2009, the overall prevalence of both species among school-aged children (SAC) was estimated at 23.5%, with 21% attributed to urogenital schistosomiasis.^14^ Since 2010, in accordance with WHO guidelines, Togo has been implementing PC with praziquantel based on the prevalence in each subdistrict.^14^ This PC has led to a substantial reduction in prevalence nationwide, dropping from 23.5% to 5% for both species and from 21% to 4.2% for *S. haematobium* between 2009 and 2015.^3 14^

Despite the overall reduction, certain areas, particularly in the Plateaux region where study was conducted, continue to exhibit schistosomiasis hotspots. Prevalence surveys conducted in this region have shown that, while nationwide prevalence has decreased, localised rates remain high due to ecological and sociocultural factors.^14^ In this region, the average prevalence of *S. haematobium* infection remains high, estimated at 7.3%, ranging from 11.3% in Est-Mono district to 15.7% and 29.0%, respectively, in Anié and Ogou districts (Unpublished data, Ministry of Health, Togo, 2015)^20^ indicating persistent transmission pockets that require targeted interventions.

This persistence can be attributed to several factors, including variations in environmental conditions such as water sources and snail habitats, as well as cultural practices like agricultural activities and water contact behaviours. Additionally, inadequate access to PC and other control measures in these settings may contribute to the sustained transmission of the disease.

This study aims to provide comprehensive evidence for a detailed epidemiological understanding of *S. haematobium* distribution among SAC following multiple rounds of PC, focusing on the associated risk factors to inform targeted interventions to address these persistent hotspots of high endemicity. The study was conducted as part of a broader multicountry initiative, the Schistosomiasis Oversampling Study.^21^

## Methods

### Study design and population

The cross-sectional survey in households was conducted from July to August 2022, involving SAC of both sexes, aged between 5 and 14 years, in the Plateaux region of Togo. In the study villages, SACs included those who had been living for the past 6 months in the selected households, for whom parents/guardians signed the consent form, and who provided their assent to participate. Those who received antiparasitic treatment within the past 6 months prior to the study or who were unwell on the day of the survey were not enrolled.

### Inclusion and non-inclusion criteria

#### Inclusion criteria

Participants must have lived in the village where the survey is conducted for at least 6 months. They must also be willing to provide informed consent. In addition, any small settlements and hamlets with a population of at least 40 individuals qualify as Potential Implementation Units for this study.

#### Non-inclusion criteria

Participants under the age of 5 or over the age of 14 will not be eligible for inclusion. Those who have taken antiparasitic medication within the previous 2 weeks or are unwilling or unable to provide consent are also excluded. Additionally, individuals who are unwell on the day of the survey will not be included, nor will large urban areas with populations exceeding 100 000.

### Study area

**Figure 1:**
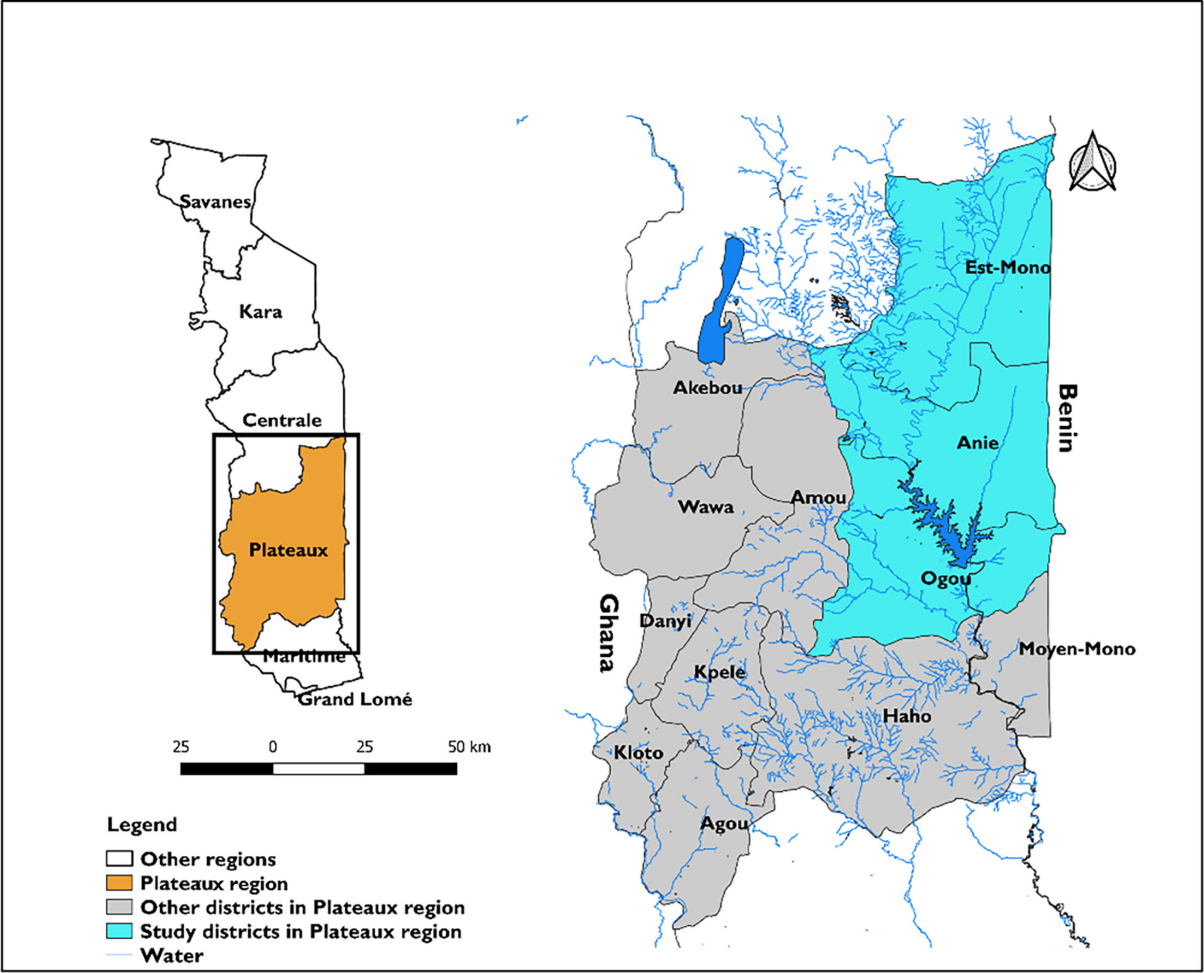
Map of Togo, showing the three districts of the community-based schistosomiasis oversampling survey, Plateaux region, Togo 2022.

This study was conducted in Est-Mono, Anie and Ogou districts in the Plateaux region of Togo (figure 1). These rural districts have a total area of approximately 5012 km² and a population of around 598 085 inhabitants according to the 2022 census.^22^ Although agriculture is the main economic activity of this population, fishing-related activities also play an important role due to the proximity of water bodies such as the Mono, Anié and Ogou rivers, as well as several small lakes and ponds in the region. These water bodies also provide recreational opportunities such as swimming and boating. However, the water, sanitation and hygiene (WASH) indicators measuring access to clean WASH are lower in these districts. According to UNICEF, in 2017, only 42% of the population in Anié had access to improved water sources, which increased to 56% and 70%, respectively, in the districts of Est-Mono and Ogou. Regarding sanitation, only 17% of the population in Anié had access to improved sanitation facilities, compared with 21% in Est-Mono and 28% in Ogou.^23^ These low indicators have significant impacts on the health and well-being of the school-age population.^23^ The three districts were included in this study based on their fulfilment of the predefined criteria, which were as follows: (1) representation of the archetype of small perennial water bodies and artificial lakes; (2) contiguity to each other; (3) moderate or high baseline prevalence of *S. Haematobium*, with a prevalence in the most recent epidemiological survey above 5% and (4) safe and accessible for fieldwork purposes.

### Sampling design

This study used a two-stage stratified random sampling ‘modified segment’ approach.^24 25^ The stratification of districts was based on key characteristics including population density, proximity to water bodies and historical prevalence data of schistosomiasis to guarantee representativeness.

### Primary sampling units

A detailed inventory of all villages with their estimated populations per district was compiled. Larger villages were disaggregated into smaller subunits to consider both population size and geographical distribution, thus ensuring the manageability of each unit for sampling purposes. Stratification of primary sampling units (PSUs) was conducted based on population and environmental risk factors, and 40% of all PSUs were randomly selected to capture spatial variations and prevalence distinctions within the region.

### Selection of segments

Within the chosen PSUs, villages or subunits were subdivided into segments, typically comprising around 50 households each. A random number was assigned to each segment, and using a random number generator, one segment per PSU was selected. Following this selection, all households within the segment were identified.

### Systematic household sampling

After the enumeration of households within each segment, a systematic sampling approach was implemented to ensure representativeness. Predetermined sampling lists were used, enabling the selection of every nth household based on the total number of households in the segment to attain the desired sample size.

### Inclusion of SACs

All SACs from the selected households who met the predefined inclusion criteria were invited to participate. The minimum target sample size for SACs was set at 36 per segment. In situations where children were absent during the survey, households were revisited by the survey team to enhance participation rates.

### Local assistance and revisits

A local guide was enlisted in each village to aid in the identification and revisiting of selected households, ensuring thorough inclusion and reducing non-response bias. This proactive measure aimed to prevent the omission of eligible SACs in the sampling.

### Data collection

Direct interviews with household heads or the community workers were used to collect geographical data (Global Positioning System (GPS) coordinates), WASH information, as well as information related to the economic level of the selected households. Additionally, SACs from these households provided information, with the help of a guardian or by responding individually, on their fishing and swimming practices and school attendance. Furthermore, each SAC that consented was asked to provide a urine sample for the detection *of S. haematobium ova*.

The questionnaire used during data collection was structured into several key sections, including 60 questions in total: Survey Details, Household Information, Equity Questions, WASH, Household Economic Practices, Individual Data Collection and Sample Collection. For more detailed information on the survey questions, please refer to online supplemental document.

The interviews were conducted by trained investigators to ensure consistency and reduce interviewer bias. Investigators underwent comprehensive training on the use of data collection tools, interview techniques and study protocols. Data collection was carried out using Secure Data Kit software on smartphones, with data uploaded and managed on the Standard Data platform (www.datastandard.co).

### Survey validation and quality control

This survey is part of a multicentric study, with the pilot survey conducted in Kenya, which served as the basis for refining subsequent surveys in Togo.^21^ Prior to implementation, a 1-week training session was held, during which the survey team was trained on interview procedures and biological sample collection. Following the training, a pretest survey was conducted, and a debriefing session was held to address and correct any identified issues. The pretest survey and feedback allowed for necessary adjustments to ensure the quality and reliability of the survey before full implementation.

To maintain high-quality standards during the survey, each team was supervised by a field supervisor. The data collected were reviewed daily by two data managers, who identified errors and provided feedback for corrections. Additionally, to further ensure data accuracy, a paper-based data collection system was implemented alongside the electronic system as a backup. The survey was reviewed by a team of professionals, including epidemiologists, public health experts and data managers, ensuring that the content and procedures were appropriate for the objectives of the study.

### Sample collection

Each SAC enrolled in the study provided a urine sample. These were collected between 10:00 and 14:00 hours in a clean, transparent plastic container supplied by the survey team. Each sample was labelled with a QR code associated with a unique identification number. The children were encouraged to drink water and/or engage in physical exercise before urination to increase the volume of urine provided and to promote egg detachment from the bladder wall.^26^ The samples were stored in a cooler and immediately transported to the laboratory for analysis to prevent egg hatching and crystal formation.

### Diagnosis of urogenital schistosomiasis

Urine filtration was used for the detection and quantification of *S. haematobium* eggs. A 10 mL of each homogenised urine sample was filtered through a 13 mm diameter nylon filter with a 12 µm pore size. Once the filtration was complete, the filter containing the residues, including *Schistosoma* eggs, was carefully removed and transferred onto a clean microscope slide. A drop of Lugol’s iodine was added to the preparation then examined under low magnification.

A comprehensive scan of all fields on the slide was conducted, with slides revealing egg presence recorded as positive, whereas slides devoid of eggs were marked as negative. The infection’s intensity for the positive samples was ascertained and documented as the number of eggs per 10 mL of urine. The infection intensity was categorised as either light infection (fewer than 50 eggs/10 mL of urine) or heavy infection (more than 50 eggs/10 mL of urine). In instances where the volume of urine provided by the SACs was less than 10 mL, the egg count was adjusted accordingly.

Each urine sample had previously been subjected to a urinalysis strip test to detect the presence of haematuria, an indicator of urogenital schistosomiasis. The outcome of this test will be the subject of another article.

### Quality control

To ensure quality control, 10% of the prepared slides were randomly selected and re-examined by another experienced technician. In addition to the electronic database, the data were also recorded on physical forms, and two data managers verified the quality of the recorded data on the electronic platform every day. Any discrepancies were reported, and the data from these cases were cross-checked with the data recorded on the paper forms.

### Data processing and statistical analysis

The survey data were downloaded from the hosting platform in Microsoft Excel format. The data were processed, and children who did not provide urine samples were excluded from the final dataset. Statistical analyses were conducted using Stata V.16 software (StataCorp).

In this study, we focused on two dependent variables: urogenital schistosomiasis and intensity of infection. Urinary schistosomiasis was assessed by detecting the presence or absence of Schistosoma eggs in urine samples, and the data were divided into two groups: positive (presence of eggs) and negative (absence of eggs). Intensity of infection was considered as a continuous counting variable based on the number of eggs per 10 mL of urine.

We explored associations between infection status and individual and household occupational factors. The chosen variables were based on their potential effects on urogenital schistosomiasis according to previous studies.^27^,^32^

The selected individual-level variables included age, sex, education, fishing practice and swimming in surface water. Age was categorised as ‘5–9’ and ‘10–14,’ sex was recoded as ‘girl’ or ‘boy’. School attendance, fishing practices and playing in water in the past 5 days were coded as ‘no’ and ‘yes.’

Household-level variables included household fishing practice, washing clothes, fetching water outside the home, irrigated land cultivation, flooded land cultivation, source of drinking water and the presence of sanitary facilities. Household fishing practice, fetching water outside the home, irrigated land and/or flooded land cultivation, presence of sanitary facilities, and different sources of drinking water, including drinking public tap water, drinking borehole water, drinking rainwater, drinking surface water, and drinking well water or drinking unprotected well water, were coded as ‘no’ and ‘yes’. Finally, the location of washing clothes was coded as ‘home’' and ‘river’.

Summary statistics were used to describe categorical variables in terms of frequency with 95% CI, and quantitative variables were summarised using means and SD.

The χ^2^ test was employed to assess the association between schistosomiasis infection status and exploratory variables. For associations involving infection intensity, we applied the Mann-Whitney test for variables with two categories and the Kruskal-Wallis test for those with more than two categories, as these tests are suitable for non-normally distributed data.

To identify the potential determinants of urogenital schistosomiasis and its intensity, we employed a multilevel logistic regression model (MLRM) for infection status and a multilevel negative binomial regression model (MNBRM) for intensity.^33 34^ The choice of the negative binomial model was motivated by the presence of overdispersion in the intensity data, where the variance (75219.94) exceeded the mean (38.18), making it more suitable than Poisson regression. We considered households and villages as random effects for infection status and only the village for intensity, to account for potential data dependence due to the focal nature of schistosomiasis and the shared behavioural factors.^35^

Four regression models were fitted for each dependent variable. The first models (intercept only models without covariates) show the variation in dependent variables attributed to clusters (households and villages). Models II and III were adjusted for individual and household variables, respectively. In model IV, individual and household variables were adjusted simultaneously.

We estimated the random effects of the models using intraclass correlation coefficients (ICCs). The MLRM and MNBRM models are mixed models that include both fixed and random effects.^33 34^ Fixed effects are presented as adjusted ORs (aOR) with their 95% CI.^33^ Random effects, which capture the variability of measured parameters between clusters, are quantified using ICCs.^33 34^

For the MLRM, the ICC was directly estimated by Stata using the ‘estat icc’ command after each model estimation.

However, for the multilevel negative binomial model, Stata does not provide a direct ICC estimate. Therefore, the ICC was manually calculated using the following formula:


(36) $$ICC=\frac{\sigma_{\mu }^{2}}{\sigma_{\mu }^{2}+\varphi }$$

Where φ, an overdispersion parameter representing the amount of unobserved heterogeneity at the individual level (intersection variance) and ($\sigma_{\mu }^{2}$) is the variance of the random parameter at the group level, representing the amount of unobserved heterogeneity between clusters.^36^ The likelihood ratio (LR) test was used to measure model fit while both Akaike (AIC) and Bayesian information criteria (BIC) were used to assess fit between the different models and the data. The variance inflation factor (VIF) was used to test multicollinearity among the explanatory variables, and no variables showed high collinearity. Variables eligible for multiple logistic regression were those with p values ≤0.20 in simple logistic regression, and variables with p values ≤0.05 were considered significant associated factors.

## Results

**Figure 2:**
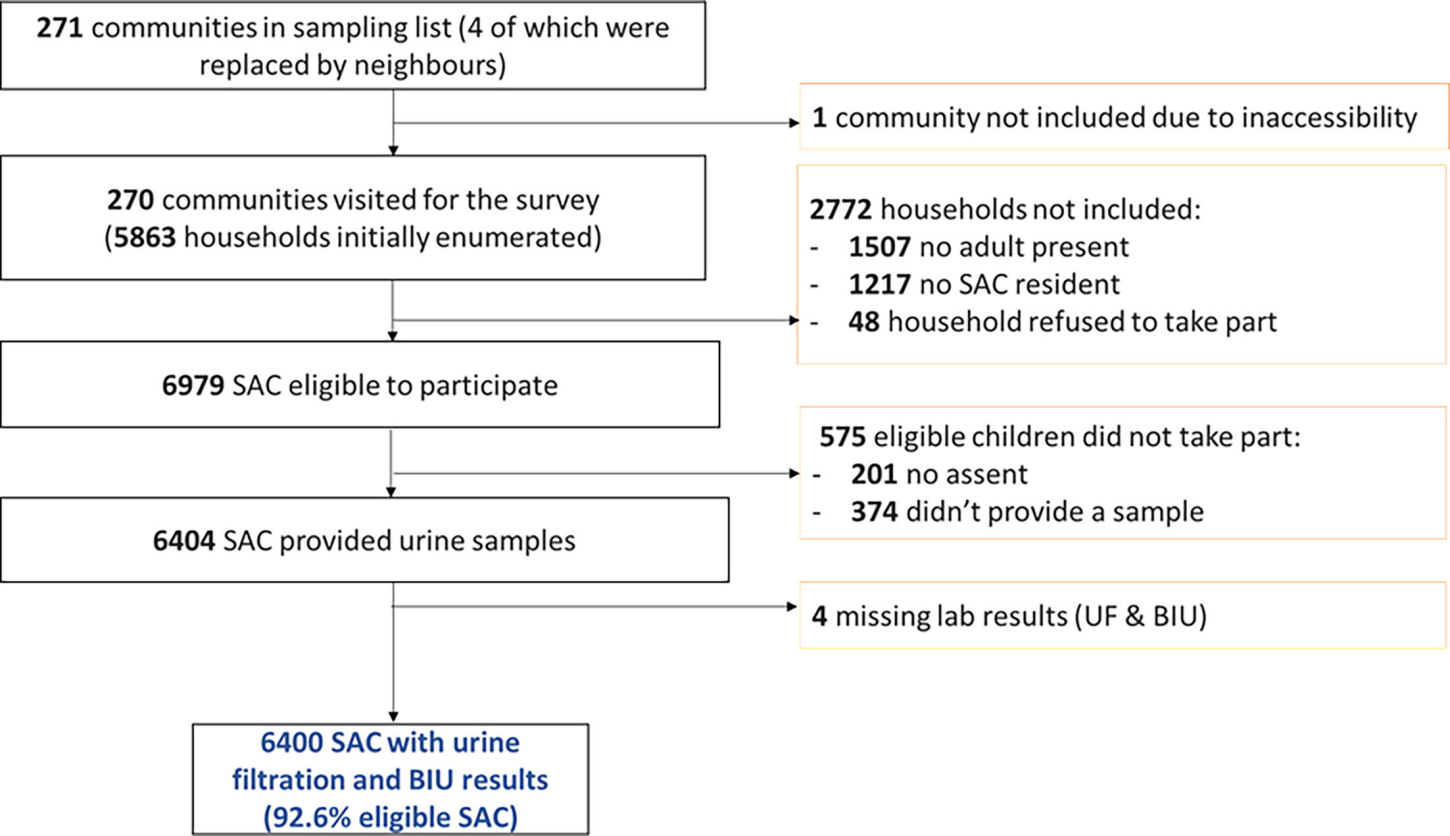
Inclusion flow chart of households and school-age children in the three surveyed districts of the Plateaux region: oversampling household survey in Togo, 2022. BIU, blood in urine; SAC, school-aged children; UF, urine filtration.

A total of 6400 school-aged children were included in this study: 1686 from Anié, 1677 from Est-Mono and 2545 from Ogou. These children came from 270 sites visited (figure 2).

### Characteristics of the study population

The median age of the 6400 enrolled SAC was 8 years (IQR: 6–11). Among them, 52.7% were boys, 84.0% attended school in the past week and 58.8% reported swimming or playing in water in the past week. In total, 67.6% of these children came from households who farmed on irrigated land and drank water obtained outside the household (91.8%). The main source of drinking water for these households was public tap (29.8%), followed by borehole water (27.5%). Most SAC (94.5%) did not have access to sanitary facilities at home (table 1).

**Table 1:** Distribution of urogenital schistosomiasis infection and heavy intensity of urogenital schistosomiasis in three districts of the Plateaux region: household oversampling survey, Togo, 2022

Variables	Total number of SACs	Urogenital schistosomiasis infection		Haematobium egg count per 10 mL		
	N (%)	Positive number (%)	P value	Mean (SD)	(Min-Max)	P value
Individual variables						
Age group (years)			<0.001			<0.001
(5–9)	4032 (63.0)	537 (13.3)		30 (217)	(0–5614)	
(10–14)	2368 (37.0)	420 (17.7)		52 (350)	(0–9688)	
Sexes			0.017			0.001
Girl	3030 (47.3)	419 (13.8)		32 (287)	(0–9688)	
Boy	3370 (52.7)	538 (16.0)		44 (262)	(0–4820)	
School attendance			<0.001			<0.001
No	1024 (16.0)	106 (10.4)		16 (144)	(0–3806)	
Yes	5376 (84.0)	851 (15.8)		42 (292)	(0–9688)	
Swimming			<0.001			<0.001
No	2638 (41.2)	192 (7.3)		10 (83)	(0–2798)	
Yes	3762 (58.8)	765 (20.3)		58 (350)	(0–9688)	
Fishing			<0.001			<0.001
No	5432 (84.9)	680 (12.5)		32 (255)	(0–9688)	
Yes	968 (15.1)	277 (28.6)		75(362)	(0–5614)	
Household variables						
Household fishing practice			<0.001			<0.001
No	5432 (84.9)	680 (12.5)		32 (255)	(0–9688)	
Yes	968 (15.1)	277 (28.6)		75 (362)	(0–5614)	
Flooded land cultivation			<0.001			<0.001
No	4235 (66.2)	386 (9.1)		26 (245)	(0–9688)	
Yes	2165 (33.8)	571 (26.4)		62 (323)	(0–5614)	
Irrigated land cultivation			<0.001			<0.001
No	2075 (32.4)	257 (12.4)		37 (317)	(0–9688)	
Yes	4325 (67.6)	700 (16.2)		39 (251)	(0–5614)	
Fetching water outside home			<0.001			<0.001
No	523 (8.2)	38 (7.1)		21 (179)	(0–3314)	
Yes	5865 (91.8)	919 (15.7)		40 (281)	(0–9688)	
Landry location			<0.001			<0.001
Home	3306 (51.7)	310 (9.4)		14 (117)	(0–3314)	
River	3094 (48.3)	647 (20.9)		64 (374)	(0–9688)	
Districts			<0.001			<0.001*
Anie	1647 (25.7)	124 (7.5)		02 (25)	(0–2798)	
Est Mono	1732 (27.1)	36 (2.1)		16 (145)	(0–679)	
Ogou	3021 (47.2)	797 (26.4)		71 (381)	(0–9688)	
Household water source						
Borehole			<0.001			<0.001
No	4638 (72.5)	798 (17.2)		39 (258)	(0–5614)	
Yes	1762 (27.5)	159 (9.0)		35 (313)	(0–9688)	
Protected well			0.001			0.002
No	5774 (90.2)	892 (15.4)		38 (277)	(0–9688)	
Yes	626 (9.8)	65 (10.4)		36 (247)	(0–3806)	
Public tap			<0.001			<0.001
No	4495 (70.2)	579 (12.9)		37 (282)	(0–9688)	
Yes	1905 (29.8)	378 (19.8)		40 (256)	(0–5614)	
Surface water			<0.001			<0.001
No	4670 (73.0)	621 (13.3)		35 (269)	(0–9688)	
Yes	1730 (27.0)	336 (19.4)		47 (289)	(0–5101)	
Rainwater			0.797			0.695
No	6377 (99.6)	954 (15.0)		38 (274)	(0–9688)	
Yes	23 (0.4)	03 (13.0)		01 (05)	(0–24)	
Unprotected well			<0.001			<0.001
No	6046 (94.5)	941 (15.6)		40 (282)	(0–9688)	
Yes	354 (5.5)	16 (4.5)		05 (44)	(0–580)	
Sanitary facility			0.011			0.011
No	6049 (94.5)	921 (15.2)		39 (278)	(0–9688)	
Yes	351 (5.5)	36 (10.3)		21 (191)	(0–3314)	

* The Kruskal-Wallis test was used for comparing the districts.

SAC, school-aged children.

The overall prevalence of urogenital schistosomiasis was 15.0% (14.10%–15.85%), with heavy intensity infection observed in 48.3% (45.1%–51.5%) of cases, an average intensity of 38 eggs/10 mL of urine and values ranging from 0 to 9688. Prevalence was highest in the Ogou district (26.4%) and lowest in the Est-Mono district (2.1%). It was also higher among boys (16.0%) and in the 10–14 age group (17.8%). Individuals engaging in swimming (20.3%) and fishing (28.5%) were more likely to be infected at the individual level. At the household level, infection was more frequent among children from households involved in fishing (28.60%), cultivation of flooded lands (26.40%), laundry in rivers (20.9%) and those using public fountains (19.8%) or surface water (19.4%) as a source of drinking water. Regarding the intensity of infection, the average number of eggs was higher in the 10–14 age group, among boys, those engaged in fishing and those washing their clothes in freshwater. All these differences were statistically significant (p<0.01) (table 1).

### Multilevel logistic regression

#### Fixed effects (association measure)

Model IV, comprehensively, demonstrates the association between individual factors and household factors associated with urogenital schistosomiasis on one hand, and the intensity of infection on the other hand. At the individual level, education and swimming in freshwater, and at the household level, district of residence, are significantly associated with urogenital schistosomiasis. For the intensity of infection at the individual level, age, sex, education and freshwater swimming are significantly associated. At the household level, consumption of public tap water and rainwater are also significantly associated.

Regarding urogenital schistosomiasis at the individual level, children aged between 10 and 14 years had a significantly higher risk (aOR=1.9; 95% CI: 1.5 to 2.4) compared with those aged between 5 and 9 years. Similarly, school children were more likely to be infected (aOR=1.8; 95% CI: 1.8 to 2.6) than those who did not attend school. In addition, freshwater bathing significantly increased the risk of infection (aOR=2.5; 95% CI: 1.7 to 3.7) compared with those who did not. Based on the household clustering, children residing in the Ogou district had a much higher risk (aOR=11.20; 95% CI: 5.2 to 24.0) than those in the Anié district. However, those living in the Est-Mono district had a very low risk of infection (aOR=0.2; 95% CI: 0.1 to 0.6).

Regarding the intensity of infection, children aged 10–14 years (incidence rate ratio (IRR)=2.9; 95% CI: 2.0 to 4.4) and boys (IRR=1.7; 95% CI: 1.2 to 2.4) were significantly more likely to be infected than their younger and female counterparts. In addition, school children had higher levels of intensity (IRR=2.4; 95% CI: 1.4 to 4.2), as were those who bathed in fresh water (IRR=4.7; 95% CI: 2.8 to 8.1). At the household level, infection intensity was significantly higher among children who use tap water as main drinking source (IRR=2.6; 95% CI: 1.2 to 5.5) and among those living in the Ogou district (IRR=28.6; 95% CI: 9.7 to 84.2) compared with those living in the Anié district. However, children residing in the Est-Mono district (IRR=0.0; 95% CI: 0.0 to 0.08) and those consuming rainwater (IRR=0.0; 95% CI: 0.0 to 0.4) had a significantly reduced risk compared with those in the Anié district and those not consuming rainwater, respectively (table 2).

**Table 2:** Factors associated with urogenital schistosomiasis and infection intensity among school-age children in the three districts surveyed in the Plateaux region: oversampling household survey, Togo, 2022

Variables	Urogenital schistosomiasis				Infection intensity			
	aOR (95% CI)				IRR (95% CI)			
	Model I	Model II	Model III	Model IV	Model I	Model II	Model III	Model IV
Fix effect results								
Individual level variables								
Age								
Age group								
(5–9)		1		1		1		1
(10–14)		1.9 (1.5 to 2.4)		1.9 (1.5 to 2.4)***		2.9 (2.0 to 4.3)***		2.9 (2.0 to 4.4)***
Sex								
Girl		1		1		1		1
Boy		1.1 (0.8 to 1.3)		1.6 (0.8 to 1.3)		1.7 (1.2 to 3.5)**		1.7 (1.2 to 2.4)**
School attendance								
No		1		1		1		1
Yes		1.7 (1.2 to 2.4)**		1.8 (1.8 to 2.6)***		2.2 (1.3 to 3.9)**		2.4 (1.4 to 4.2)**
Plays in water								
No		1		1		1		1
Nes		2.2 (1.5 to 3.2)***		2.6 (1.8 to 3.9)***		3.8 (2.2 to 6.5)***		4.7 (2.8 to 8.1)***
Fishing								
No		1		1		1		1
Yes		1.9 (1.2 to 2.9)**		1.4 (0.9 to 2.2)		1.2 (0.6 to 2.4)		0.8 (0.4 to 1.7)
Household environmental level variables:								
Household fishing								
No			1	1			1	1
Yes			2.4 (1.6 to 3.6)***	Omitted			2.1 (1.0 to 4.2)*	Omitted
Crops flooded land								
No			1	1			1	1
Yes			1.2 (0.8 to 1.7)	1.2 (0.8 to 1.7)			0.9 (0.5 to 4.1)	0.7 (0.4 to 1.3)
Washing clothes								
Home			1	1				
River			1.7 (1.1 to 2.6)*	1.5 (0.9 to 2.3)				
Water source:								
Borehold								
No			1	1			1	1
Yes			0.8 (0.5 to 1.5)	0.9 (0.5 to 1.7)			0.9 (0.5 to 1.6)	1.1 (0.5 to 2.3)
Public tap								
No			1	1			1	1
Yes			1.3 (0.6 to 1.5)	1.4 (0.8 to 2.5)			2.4 (1.0 to 5.2)**	2.6 (1.2 to 5.5)*
Rainwater								
No							1	1
Yes							0.0 (0.0 to 0.4)*	0.0 (0.0 to 0.4)*
Protected well								
No								1
Yes								2.2 (0.9 to 4.59)
Districts								
Anie			1	1			1	1
Est Mono			0.2 (0.1 to 0.6)**	0.2 (0.1 to 0.6)**			0.2 (0.0 to 0.9)***	0.0 (0.0 to 0.08)***
Ogou			9.0 (4.0 to 19.3)***	11.20 (5.2 to 24.0)***			18.6 (6.3 to 55.0)***	28.6 (9.7 to 84.2)***

* p<0.05, **p<0.01, ***p<0.001.

AOR, adjusted OR; IRR, incidence rate ratio.

#### Random effects (measure of variance) and model fit

The empty model revealed significant variation in the prevalence of urogenital schistosomiasis attributable to clustering at both the village level (σ²=8.1, 95% CI (6.0 to 11.0)) and the household level (σ²=1.8, 95% CI (1.2 to 2.6)). For infection intensity, significant variation was observed at the village level (σ²=29.6, 95% CI (21.1 to 39.2)).

For prevalence, the empty model showed that 61.5% of the total variance could be attributed to differences between villages (ICC=0.615), while 75.1% was explained by household-level differences (ICC=0.751). In the full model, incorporating both individual and household/community-level covariates, the ICC at the village level decreased to 44.6%, and at the household level to 63.5%, indicating that some variation was explained by the included covariates. For infection intensity, the village-level ICC in the empty model was 62.2%, suggesting that most of the variance in infection intensity is explained by differences between villages. In the final model, after accounting for individual and household variables, the village-level ICC decreased to 52.6%, demonstrating the role of these factors in explaining variations in infection intensity (table 3).

**Table 3:** Random effects and model fit statistics for factors associated with urogenital schistosomiasis and infection intensity among school-age children in the Plateaux Region: Oversampling Household Survey, Togo, 2022

Model parameters and fit indices	Urogenital schistosomiasis				Infection intensity			
	aOR (95% CI)				IRR (95% CI)			
	Model I	Model II	Model III	Model IV	Model I	Model II	Model III	Model IV
Random effects								
Village variance (95% CI)	8.1 (6.0 to 11.0)	7.7 (5.6 to 10.5)	4.4 (3.2 to 6.1)	4.2 (3.1 to 5.9)	29.6 (21.3 to 41.2)	28.8 (21.1 to 39.2)	20.2 (15.2 to 26.9)	18.7 (14.1 to 24.8)
Household variance	1.8 (1.2 to 2.6)	1.7 (1.1 to 2.5)	1.7 (1.1 to 2.5)	1.7 (1.2 to 2.6)				
ICC Village (%)	61.5	60.6	46.7	44.6	62.2	62.7	53.2	52.6
ICC Household (%)	75.1	74.1	64.8	63.5				
LR test*	χ2=1810.5***	χ2=1612.5***	χ2=940.3***	χ2=823.0***	χ2=968.7***	χ2=937.06***	χ2=828.9**	χ2= 803***
Wald χ2	reference	86.21***	117.0***	175.9***	reference	93.8***	152.4***	222**
Model fitness								
Log-likelihood	−1794.9	−1746.6	−1734.3	−1693.4	−7825.7	−7784.1	−7779.4	−7735.0
AIC	3595.8	3509.2	3507.0	3414.4	15 657.4	15 584.0	15 578.8	15 498.0
BIC	3616.1	3563.3	3575.0	3499.0	15 677.6	15 638.1	15 646.5	15 592
VIF					1.76			

Model I is the null model, a baseline model without any determinant variable.

Model II is adjusted for individual level variables.

Model III is adjusted for household/community level variables.

Model IV is the final model adjusted for individual and household/community level variables.

* The LR tests compare the likelihoods of target models, including random effects, to those of their corresponding null models, which do not include random effects; **<0.01; ***p<0.001.

AIC, Akaike’s information criterion; AOR, adjusted OR; BIC, Bayesian information criterion; ICC, intraclass correlation coefficient; IRR, incidence rate ratio; LR, likelihood ratio; VIF, variance inflation factor.

Model fit was assessed using log-likelihood, AIC and BIC. The model with the best fit was model IV, which controlled individual-level and household-level covariates. The VIF, a measure of multicollinearity, was 1.76 for both independent variables, which is below the recommended threshold, indicating no significant correlation between the independent variables (table 3).

## Discussion

This study aimed to explore the distribution of urogenital schistosomiasis among school-aged children in three districts of the Plateaux region in Togo and identify factors associated with infection using household-level data through multilevel modelling.^35 37^ Multilevel models are widely applied in various fields, including social sciences^38^,^42^ and have also found increasing use in risk-factor analyses, including those in infectious diseases.^43^,^45^ To the best of our knowledge, this study is the first in Togo to employ multilevel modelling to investigate *S. haematobium* risk factors. Multilevel models are particularly valuable for analysing hierarchical data frequently encountered in cross-sectional epidemiological studies with cluster sampling, as they enable the examination of covariate effects at both the cluster and individual levels.^35 37 46^ Furthermore, they facilitate the exploration of interindividual and intercluster variations while assessing the relative contributions of variables at the individual and cluster levels.^33 34^

The null models revealed significant variation in urogenital schistosomiasis prevalence among clusters, with 75.1% and 61.5% of variation attributed to household and village-level differences, respectively. This underscores the focal nature of *S. haematobium* infection. The persistence of these variations after adjusting for covariates highlights the relevance of environmental and household factors. Infection intensity also varied substantially at the village level (ICC 62.2%), consistent with the understanding that schistosomiasis is often a highly focal disease,^6 10^ highlighting the role of environmental and community factors.^47 48^

The decrease in ICCs in the full models (44.6% for villages and 63.5% for households) suggests that a portion of the variation is explained by household and village characteristics, though unmeasured factors likely remain influential. These findings emphasise the importance of hierarchical models in assessing risk factors.^33^ These results suggest that interventions focusing on households and communities may be more effective than uniform national strategies. Future research should focus on identifying specific factors contributing to these variations to refine prevention strategies.^49 50^

This study demonstrates the value of hierarchical regression in analysing hierarchical survey data, providing a nuanced assessment of individual, household and community-level influences on urogenital schistosomiasis transmission and control.^33 35^ In the districts of Est-Mono, Anie and Ogou, the average prevalence was 2.1%, 7.5% and 26.4%, respectively. These results indicate a slight decline compared with the prevalence rates for each district in the 2015 impact assessment, which were 11.3%, 15.7% in the Anié district and 29.0%, respectively (unpublished data, Ministry of Health, Togo, 2015^20^). This reduction in prevalence could be attributed to the implementation of control programmes, such as mass treatment campaigns and efforts to improve WASH initiatives.^3 48^

The difference in average prevalence between districts can be explained by various factors, including variations in sanitation, the presence of stagnant waters that support the life cycle of parasites, and the proximity and behaviours of children towards these water sources,^51 52^ and the difference in prevalence between years may be related to the implementation of seasonal chemoprophylaxis.^3 14^ The quality of chemoprophylaxis is strongly associated with the reduction of schistosomiasis prevalence^53^; properly implemented PC, according to the appropriate frequency, significantly reduces schistosomiasis prevalence.^14 54^ Further investigation into MDA effectiveness and adherence is necessary, particularly in regions with persistent prevalence.

Assessment of risk factors revealed a significant association between urogenital schistosomiasis and schistosomiasis infection intensity and several individual factors, such as age, sex, education and swimming. Children aged between 10 and 14 years were nearly twice as likely to have urogenital schistosomiasis and nearly three times as likely to have several infections compared with those aged 5–9 years. For sex, boys were 1.2 times more intense than girls. Our results are consistent with previous studies in sub-Saharan Africa^55 56^ and other parts of the world.^57^ For example, a study in Nigeria found that children aged 10–14 years had 1.68 times higher odds of infection compared with younger children.^56^ Similarly, research in Ivory Coast showed that older children (10–15 years) had significantly higher infection rates than younger age groups.^55^ Studies in other parts of the world, such as Yemen, also reflect this trend, where increased water exposure among boys led to higher infection rates^57^ Additionally, these studies reported higher infection intensities in boys compared with girls, aligning with our findings of 1.2 times higher intensity in boys.

These results may be explained by the fact that children aged 10–14, and especially boys, are more likely to be involved in activities that put them in greater contact with cercarial-infested waters such as irrigated cropping, fishing and swimming and are thus at greater risk of infection with *S. haematobium*.

Children who bathed in freshwater were 2.6 times more likely to develop urogenital schistosomiasis and about five times more likely to have a heavy intensity infection than those who did not bathe in freshwater. These results are consistent with previous studies that have also shown a significant association between urogenital schistosomiasis and swimming.^27 30 48 56 58^ Indeed, children who engage in activities such as swimming, washing in water bodies infested with cercariae and living in flooded areas face heightened susceptibility to *S. haematobium* infection. It is imperative to prioritise awareness-raising efforts regarding these risky behaviours to mitigate disease transmission and bolster control measures against schistosomiasis.

Also, the analysis revealed that using public tap water was associated with infection intensity 2.6 times higher. Conversely, using rainwater reduces the risk of high-intensity urinary schistosomiasis infection compared with those who did not use it.

Although these results may seem unexpected, they underscore the potential of rainwater as a cleaner and safer source of water, which could contribute to reducing exposure to urinary schistosomiasis, if collection and storage methods are secure and hygienic.^59 60^ However, it is important to note that this correlation may not necessarily imply causation. The observed association might indicate that the source of drinking water is closely related to other measured or unmeasured risk factors for schistosomiasis. Regarding tap water use, which was associated with an increased risk of intense infection, this could be attributed to the quality of treatment of water distributed by the taps, especially water sourced from rivers.^48 60^ This result is consistent with previous studies that have also shown that access to clean and safe drinking water is essential to reduce the prevalence of schistosomiasis.^4858^,^60^

Our research highlights the importance of raising awareness among communities in regions affected by schistosomiasis about water-related practices, especially swimming and fishing, to effectively combat the disease.^61 62^ This is supported by findings from Balola *et al*^63^ and Torres-Vitolas *et al*,^64^ which emphasise the impact of behaviour change interventions and WASH strategies on reducing schistosomiasis transmission. These studies demonstrate how integrating community-based efforts with improved access to clean water and sanitation facilities can significantly reduce infection rates and contribute to long-term disease control.

Parasitological analysis of public fountain water, sourced from river sources, is essential to ensure its quality.^65^ This strategy, explained by the mode of transmission of schistosomiasis, requires a combined approach of PC with education and awareness campaigns. These campaigns aim to provide information regarding the role of contaminated water in the transmission of the disease and promote behavioural changes to mitigate the rates of infection. Moreover, it is crucial to integrate the promotion of access to safe drinking water into the schistosomiasis control strategies within communities,^65^ thus reducing contact with contaminated water sources and consequently the risk of exposure to infection.^48 66^ This holistic approach addresses the direct causes and vectors of transmission, proving essential for an effective fight against this disease.

Further findings revealed a significant correlation between school attendance, residence in the Ogou district, and an increased risk of infection by *S. haematobium*. School-going children were about twice as likely to suffer from urinary schistosomiasis with a heavy intensity infection. Although the link between schooling and an increased risk of infection may seem counterintuitive, it can be explained by the inevitable exposure of students to infested waters. This exposure comes either through swimming practices, daily crossings of water bodies to reach school, or the consumption of non-potable water on school premises. These findings suggest that educational establishments offer an ideal platform for deploying targeted health education programmes,^67^ aiming to raise awareness among students about the dangers of schistosomiasis and effective preventative practices. In this context, we recommend the inclusion of educational modules on water management and personal hygiene in school curricula, as well as the installation of safe drinking water systems in schools. Previous studies have demonstrated the effectiveness of such interventions in reducing the prevalence of schistosomiasis among school populations.^48 59 68 69^

Looking at the results for each district, we found that residents of Ogou district were 11 times more likely to contract schistosomiasis and had an infection intensity 28 times higher than those in Anié district. In contrast, children from Est-Mono district had an eighty times lower likelihood of infection, with no significant infection intensity observed. The most plausible explanations for the difference in schistosomiasis prevalence and intensity by district would be, first, the presence of environmental, social and biological risk factors that promote a higher infection rate in the Ogou district.^70^ Second, this difference could be attributed to the quality of mass treatment implementation in this district, as treatment quality and high treatment coverage for all age groups at risk are crucial for the effectiveness of schistosomiasis control. Lastly, the substantial burden of urogenital schistosomiasis in the Ogou district highlights the imperative need for a thorough reassessment of both the effectiveness and the acceptance of praziquantel chemotherapy within this particular focus area. This has been emphasised by Yamaney in Egypt in 2017^71^ and by Okoyo *et al* in Kenya in 2021,^72^ who reported a positive correlation between treatment coverage and reduction in helminth infection prevalence. Additionally, the situation in the Ogou district may be linked to recurrent reinfection due to activities involving continuous contact with contaminated waters.

Regardless, it is important to understand these differences in risk to implement effective prevention measures tailored to each area based on the dynamics of transmission, especially in high-risk areas.^73 74^ This will help eliminate this parasitic disease as a public health problem in endemic countries. These measures may include hygiene education programmes, provision of clean water, efforts to eliminate contaminated water sources, as well as targeting the intermediate mollusc hosts of these parasites.^48^

### Methodological limitations

Despite the strengths of this study, several methodological limitations warrant acknowledgement. First, although the community-based sampling method aimed to mitigate the exclusion of non-schooled children, certain remote populations may still be under-represented, potentially affecting generalisability. Additionally, the cross-sectional design limits the ability to establish causality between identified risk factors and urogenital schistosomiasis.

Self-reported behavioural data may introduce recall or social desirability bias, particularly regarding personal hygiene or water access. To reduce this, multilevel analysis was used, though the absence of specific contextual variables, such as water management practices, may constrain the model’s comprehensiveness.

Finally, the findings, while insightful for the Plateaux Region, may not be fully generalisable to other regions with differing environmental or socioeconomic contexts. Replication in diverse settings is necessary to confirm the broader applicability of these results.

## Conclusions

This study provides a detailed epidemiological assessment of urogenital schistosomiasis among school-aged children in the Plateaux Region, with insights into associated risk factors following multiple rounds of PC. The findings underscore persistent hotspots, notably in the Ogou district and highlight behaviours such as freshwater bathing as significant infection risks. Additionally, the context of household and village clustering largely explains the infection’s dynamics and intensity, suggesting that local transmission is influenced by community and family groupings, which should be considered in future intervention strategies.

The results suggest that targeted interventions are needed to promote access to safe drinking water, raise awareness of transmission practices such as swimming, fishing and improve coverage of PC. Such measures could help reduce the burden of schistosomiasis in these communities.

In the future, it will be essential to develop integrated control strategies that consider variations in infection risk between districts. By adapting prevention measures to local transmission dynamics, it will be possible to optimise the effectiveness of interventions. Additionally, implementing educational approaches in schools should be considered to increase awareness of schistosomiasis and preventive behaviours among the young population.

Finally, continuous research on urogenital schistosomiasis is crucial to monitor disease trends and guide public health policy. By integrating insights with context-sensitive interventions, we can make significant strides towards the goal of eliminating schistosomiasis as a public health concern in Togo.

## Supplementary material

10.1136/bmjph-2024-001304 online supplemental file 1

## Data Availability

Data are available on reasonable request.
